# Emerging trends and research foci in autophagy of pancreatic cancer: a bibliometric and visualized study

**DOI:** 10.3389/fonc.2023.1220435

**Published:** 2023-06-20

**Authors:** Linlin Fan, Zhiyong Wei, Lili Liu, Xiaojie Qi, Hong Yu

**Affiliations:** ^1^ Dalian Medical University, Dalian, Liaoning, China; ^2^ Department of Pathology, Linyi People’s Hospital, Linyi, Shandong, China; ^3^ Department of Pathology, The Affiliated Taizhou People’s Hospital of Nanjing Medical University, Taizhou, Jiangsu, China

**Keywords:** autophagy, pancreatic cancer, tumor microenvironment, bibliometric study, pancreatic stellate cells, ferroptosis

## Abstract

**Objective:**

The purpose of this study was to analyze the trends by year, country, institution, journal, reference and keyword in publications on the autophagy of pancreatic cancer (PC) and to predict future research hotspots.

**Methods:**

The Web of Science Core Collection was used to search for publications. The contributions of various countries/regions, institutes, authors, identified research hotspots, and promising future trends were analyzed using the VOSviewer1.6.16 and CiteSpace6.6.R2 programs. We also summarized autophagy relevant clinical trials of PC.

**Results:**

A total of 1293 papers on the autophagy of PC published between 2013 and 2023 were included in the study. The average number of citations per article was 33.76. The China had the most publications, followed by USA, and a total of 50 influential articles were identified through co-citation analysis. Clustering analysis revealed clusters of keywords: metabolic reprogramming and ER stress, mTOR-mediated apoptosis, extracellular trap as the most concerned clusters. The co-occurrence cluster analysis showed pancreatic stellate cell, autophagy-dependent ferroptosis, autophagy-related pathway, metabolic rewiring, on-coding RNA as the highly concerned research topics in recently.

**Conclusion:**

The number of publications and research interest have generally increased over the past few years. The China and USA have made prominent contributions to the study of the autophagy of PC. The current research hotspots mainly focus not only on the related modulation, metabolic reprogramming, ferroptosis of tumor cells themselves, but also on tumor microenvironments such as autophagy associated pancreatic stellate cells and new treatments targeting autophagy.

## Introduction

Pancreatic cancer (PC) is a common digestive system tumor and has become the fourth cause of tumor-related death in worldwide (1). By 2030, pancreatic cancer could be one of the top three causes of cancer related deaths in the United States (2). PC is difficult to diagnose at the early stage, more than 80% of PCs are already in the advanced stage when detected, and the opportunity for surgical resection is often missed due to local infiltration and metastasis (3). As a result, there is an increasing interest in pancreatic cancer of the pathogenesis and treatment strategies as a research target due to its high incidence and poor prognosis. In recent years, researchers have found that as a cancer progresses, the body can produce a variety of responses to tumors, such as apoptosis,pyroptosis, ferroptosis, cuprotosis,disulfidptosis. and autophagy (4–9). Autophagy is a process of engulfing cytoplasmic proteins or organelles and encapsulating them into vesicles, then fusing them with lysosomes to form autolysosomes and degrade their encapsulated contents, thereby fulfilling the metabolic needs of the cell itself and the renewal of certain organelles (9). Autophagy can be seen in the physiological and pathological processes of the body and a myriad of studies demonstrate important protective roles for autophagy against disease (10). However, For a long time, humans understood autophagy has not been fully elucidated and now believe that the recycling capacity of autophagy arguably makes it a growth pathway and provides an internal source of nutrients for growing cancer cells (11). Under harsh conditions with disturbed vasculature and hypoxic tumor microenvironment (TME), this internal nutrient source can play a key role in tumor cell survival (12, 13), the role of autophagy in PC is complex and affect the occurrence, development, invasion, and metastasis of PC (14).

Institutions and researchers aiming to target the relationship between pancreatic tumor cells and the autophagy and provide new ideas for the clinical treatment of PC. One such strategy is hydroxychloroquine, which prevent autophagy. Autophagy may prevent normal cells from developing into tumor cells, but in cancer it may also protect tumor cells by destroying anticancer drugs or substances taken up by them (15–18). For this reason, Anti-autophagy therapy treatment has been studied as a new methods of cancer treatment, Given the complexity and heterogeneity of pancreatic cancer in humans, the specific effect needs to be confirmed by further clinical studies. Recently, due to increasing interest in the autophagy of PC, hundreds of academic articles have been published on this topic. Therefore, the publication trends of this research area urgently need to be summarized to serve as a reference for future studies.

**Figure 1:**
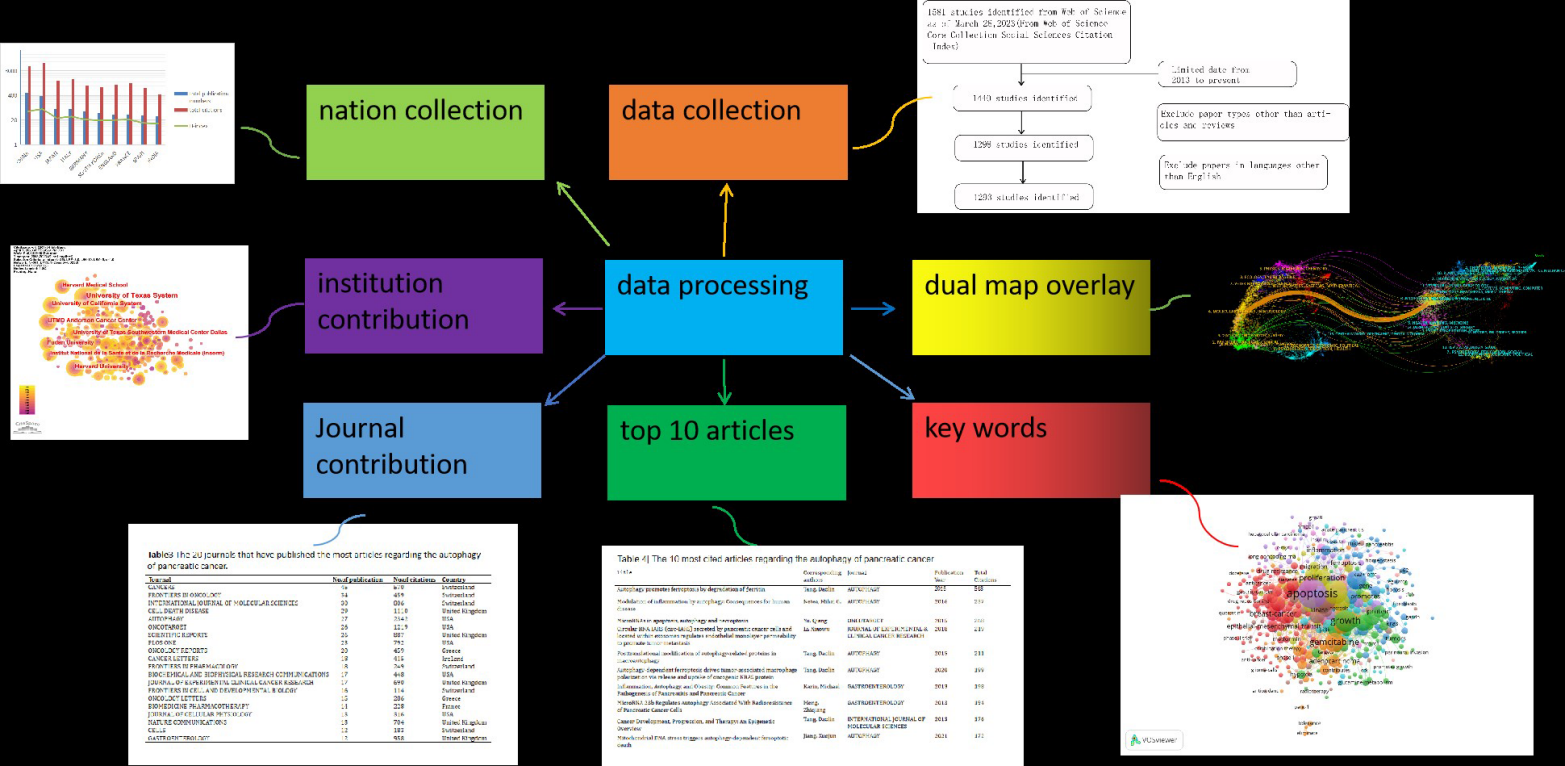
We searched the Web of Science Core Collection (WOSCC) on autophagy of PC and performed a visualization analysis from the retrospectives of countries, institutions, authors, journals, citations and keywords to predict future research hotspots in this field.

At present, we have entered the era of big data in scientific research. In the face of a large number of scientific research data, how to effectively identify and rank important research results in order to study the background and trend of the development of a problem has become a trend that attracts the attention of researchers. And because published and ongoing research has a considerable impact on the clinical outcomes of pancreatic cancer patients, it is crucial to comprehend the messages that come from this pool of accumulated literature. In this context, the bibliometric method may be a way to solve these problems. Bibliometric is a commonly used method to summarize trends in publications and analyze the literature in a certain field. This approach relies on research methods such as statistics to visually summarize research progress in a field, predict research hotspots, and furthermore, it can assess the progress made thus far in both qualitative and quantitative terms, resulting in a better understanding and description of the dynamics of scientific findings in each research area (19). In recent years, bibliometric has also been applied in multiple diseases (20–22). Although some studies have analyzed the developmental trend of targeted therapies,immunotherapy,Neoadjuvant therapy,tumor Microenvironment for PC by means of bibliometric (23–26), nobibliometric studies or visualization analyses of the autophagy of PC have as yet been reported. The WOSCC database contains over 15,000 influential high-quality journals from all over the world and is regarded as one of the most comprehensive, systematic, and authoritative databases. According to previous studies, papers in the Web of Science Core Collection (WOSCC) can represent the status of medical science (27). In the present study, we used bibliometric statistics to comprehensively analyze the literature related to the autophagy of PC by searching the Web of Science Core Collection (WOSCC). We performed a visualization analysis on the number of publications, citations, and research trends by country, author, institution and keywords using VOSviewer,CiteSpace (28) and other software to predict future research hotspots in this field (**Figure 1**).

## Materials and methods

### Data sources and search strategies

Utilizing the WOSCC database, we conducted a comprehensive search of the literature on the autophagy of PC between 2013 and March 2023. To prevent errors caused by database updates, all literature searches were completed on March 28, 2023.

Inclusion Criteria was as followed:

(1) Articles published in the last decade, between 2013-2023.
(2) English literature included in the Web of Science core anthology.
(3) The article type is article or review
(4) Literature related to pancreatic cancer or pancreatic adenocarcinoma and autophagy.

Exclusion Criteria was as followed:

(1) Articles published before January 1, 2013
(2) Literature not included in the core anthology
(3) Literature published in non-English language
(4) Conference literature and other non-monographs and review literature
(5) Literature not related to pancreatic cancer or autophagy

All information was exported in.txt format. The raw data can be found in the **Supplemental File** (**Supplemental File 1**).

### Bibliometric analysis

The WOSCC is commonly used in research on publications in the field of bio-medicine. We used the WOSCC to describe the characteristics of the literature and analyzed the distribution of publications across various countries/regions, regions, institutions, authors, and journals. The RRI, H index, were extracted from WOSCC. Relative research interest (RRI) is based on the number of publications in a field in a given year among the number of publications in all fields in that year. The H-index is a bibliometric measure that combines quantity (publications) and impact (citations). It allows us to objectively characterize the scientific output of a researcher. The H-index may be superior to other commonly used bibliometric measures, such as the total number of papers published (NP) and the total number of citations garnered (NC), as a representative measure of individual scientific achievement. CiteSpace software (from 6.2. R2 64-bit, Chaomei Chen, Drexel University, USA) was used to analyze the screened literature, and a cocitation analysis was performed on authors, countries/regions, and institutions. Cluster and co-occurrence analyses were conducted on keywords, and from which the strongest citation bursts of the keywords and references were derived. The literature was then subjected to analysis by dual-map overlays. VOSviewer (a program operated by the Center for Science and Technology Studies at Leiden University that is used to create data maps) was used for the co-occurrence analysis of countries, organization and keyword clusters.

We searched ClinicalTrials.gov (https://clinicaltrials.gov/) and International Clinical Trials Registry Platform(ICTRP,https://www.who.int/clinical-trials-platform)to summarize clinical studies on the autophagy of PC. The search strategy included the following: Condition or disease = Pancreatic Cancer; Other terms = autophagy; Study type = Interventional Study (Clinical Trials). A total of five studies regarding the autophagy of PC were identified by manual screening.

## Results

### Annual publication number and trend

**Figure 2:**
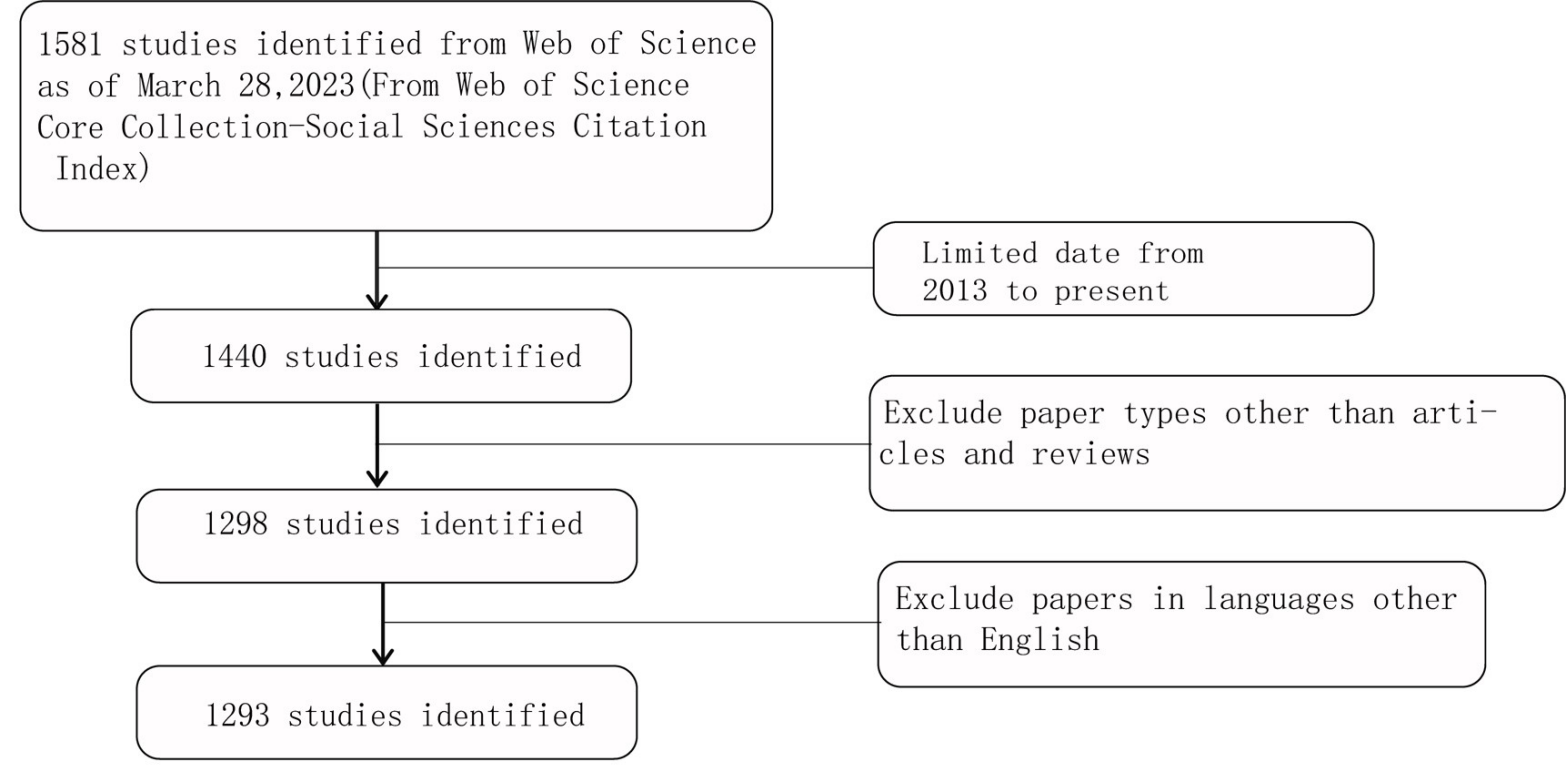
Flow chart of the screening process for research on autophagy in pancreatic carcinoma.

**Figure 3:**
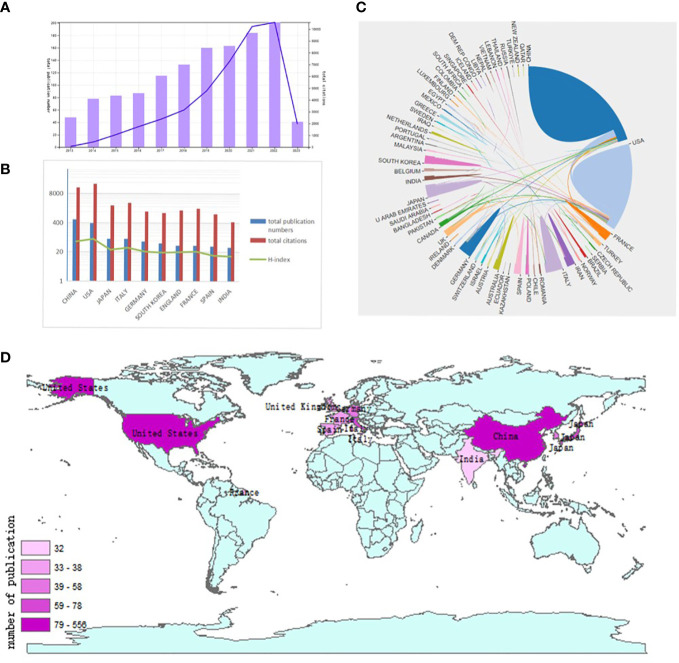
Articles related to the autophagy of pancreatic cancer published worldwide and by country/region. **(A)** The total publication number and RRI. **(B)** The total publication number, total citations, and H-index of the 20 most productive countries/regions. **(C)** Collaboration between countries/regions based on https://bibliometric.com. **(D)** The publication density map.

The flow chart shows the process of data processing (**Figure 2**). According to the inclusion criteria, a total of 1293 papers in the WOSCC database were included (the original data can be found in **Supplement File 2**). The annual publication number increased over time. A total of 43646 citations and an average of 33.76 citations per paper were noted. The H-index was 95. The number of publications on the autophagy of PC was highest in 2022 (**Figure 3A**).

### Contribution by country

A total of 65 countries/regions published studies of the autophagy of PC. China has the greatest number of publications (556 publications, accounting for 43% of all publications worldwide; a total of 14849 citations, with an average of 26.71 citations per paper and an H-index of 58, followed by the USA (384 publications, accounting for 29.7%, a total of 21509 citations, with an average of 56.01 citations per paper and an H-index of 76) and Japan (78 publications, accounting for 6.03%; a total of 2355 citations, with an average of 30.19 citations per paper and an H-index of 25)(**Figure 3B**). Although the total publication number of China were much higher than the USA,the total number of citations of papers and the H-index from the USA were much higher than those of papers from other countries/regions. The number of publications of Japan is much less than China, however, the average number of citations of each paper was 30.19, ranking second. Although Italy had fewer publications than Japan, it had a higher total number of citations, ranking third. The USA and China cooperated closely with other countries/regions (**Figures 3C, D**).

### Contribution by institution

**Figure 4:**
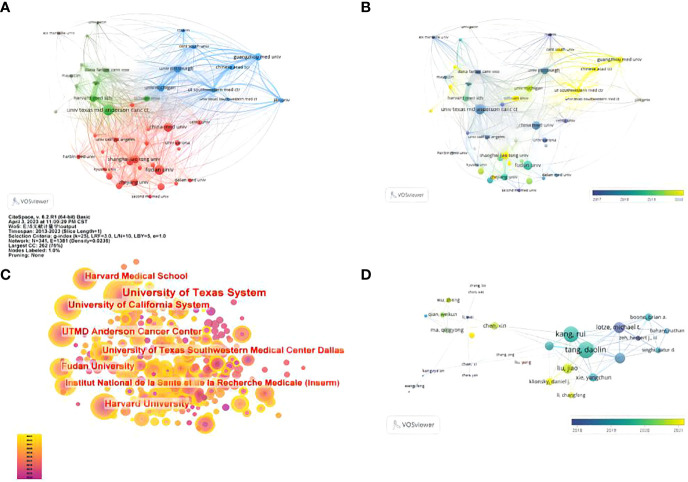
Contributions of institutions and authors to publications on the autophagy of pancreatic cancer. **(A)** The publication number network of institutions drawn by VOSviewer. **(B)** Overlay visualization of publication number of institutions drawn by VOSviewer. **(C)** Network of institutions drawn by CiteSpace. **(D)** Co-authorship cited authors (circle size represents the number of citations). Drawn by VOSviewer.

A total of 1597 institutions worldwide participated in research in this field. VOSviewer was applied to analyze the institutional citation network. Active institutions were defined as those with no fewer than 10 publications and no fewer than 100 citations. A total of 46 such institutions were identified (**Figures 4A, B**). Among them, the University of Texas M.D. Anderson Cancer Center had the most publications and highest total number of citations (**Figure 4C**). Of the 20 institutions with the most publications, most were in the USA (eleven institutions), followed by China (four institutions) and Germany (two institutions) (**Table 1**).

**Table 1:** The 20 institutes with the most publications on autophagy of pancreatic cancer.

Organization	Countries	No.of Publications	No.of Citations
univ texas md anderson canc ctr	USA	39	3990
univ pittsburgh	USA	26	2223
harvard med sch	USA	25	2199
guangzhou med univ	PEOPLES R CHINA	24	2099
univ michigan	USA	24	2525
chinese acad sci	PEOPLES R CHINA	19	766
dana farber canc inst	USA	18	2766
ut southwestern med ctr	USA	17	910
cent south univ	PEOPLES R CHINA	15	425
jilin univ	PEOPLES R CHINA	15	804
nyu	USA	15	2268
harvard univ	USA	12	1925
univ calif san francisco	USA	12	736
george mason univ	USA	10	777
canc res uk beatson inst	UK	9	802
hop europeen georges pompidou	France	8	681
univ glasgow	UK	8	694
karolinska univ hosp	Sweden	7	604
brigham & womens hosp	USA	5	707
univ paris	France	5	397

### Author contributions

A total of 8073 authors were listed for the 1293 included papers, and the average number of authors per paper was 6.24. Among the 10 authors with the most publications, seven were from the USA, and the other three were from France, China and ITLAY respectively (**Table 2**). The most published authors from the USA were from University of Texas Southwestern Medical Center Dallas. We used VOS viewer to analyze the citation network of the authors, and those with more than 50 citations were defined as key researchers. A connection represents cooperation between authors, and the circle size represents the number of citations of an author. Donadelli Massimo had the most citations at 1134 and an average of 87.2 citations per paper (**Figure 4D**)

**Table 2:** The top 10 authors who have contributed the most publications on autophagy of pancreatic cancer.

Author	Publications	Citations	Affiliation	Country
tang, daolin	25	268	UT Southwestern Med Ctr	USA
kang, rui	24	439	UTMD Anderson Cancer Center	USA
kimmelman, alecc.	16	95	NYU	USA
lotze, michael t.	15	213	Univ Pittsburgh	USA
donadelli, massimo	13	1134	Univ Verona	ITLAY
zeh, herbert j.	12	115	Univ Pittsburgh	USA
liu, jiao	11	60	Texas A&M Hlth Sci Ctr	USA
boone, brian a.	10	126	West Virginia Univ	USA
chen, xin	9	306	Guangzhou Med Univ	PEOPLES R CHINA
kroemer, guido	9	153	Univ Paris	FRANCE

### Journals publishing research on the autophagy of PC

A total of 383 journals published papers related to the autophagy of PC between 2013 and March,2023. The data were analyzed using VOSviewer. A total of 21 journals had more than five relevant publications. Among the top 20 journals in terms of the number of publications, CANCERS had the highest number of publications at 45(**Table 3**). Six of these 20 journals were published in the Switzerland, followed by USA and United Kingdom (five journals). Autophagy had the highest average number of citations for each paper at 86.74, followed by Gastroenterology and Nature Communications.

**Table 3:** The 20 journals that have published the most articles regarding the autophagy of pancreatic cancer.

Journal	No.of publication	No.of citations	Country
CANCERS	45	570	Switzerland
FRONTIERS IN ONCOLOGY	34	459	Switzerland
INTERNATIONAL JOURNAL OF MOLECULAR SCIENCES	30	806	Switzerland
CELL DEATH DISEASE	29	1110	United Kingdom
AUTOPHAGY	27	2342	USA
ONCOTARGET	26	1219	USA
SCIENTIFIC REPORTS	26	887	United Kingdom
PLOS ONE	23	792	USA
ONCOLOGY REPORTS	20	459	Greece
CANCER LETTERS	18	415	Ireland
FRONTIERS IN PHARMACOLOGY	18	249	Switzerland
BIOCHEMICAL AND BIOPHYSICAL RESEARCH COMMUNICATIONS	17	448	USA
JOURNAL OF EXPERIMENTAL CLINICAL CANCER RESEARCH	17	690	United Kingdom
FRONTIERS IN CELL AND DEVELOPMENTAL BIOLOGY	16	114	Switzerland
ONCOLOGY LETTERS	15	286	Greece
BIOMEDICINE PHARMACOTHERAPY	14	228	France
JOURNAL OF CELLULAR PHYSIOLOGY	13	316	USA
NATURE COMMUNICATIONS	13	704	United Kingdom
CELLS	12	183	Switzerland
GASTROENTEROLOGY	12	958	United Kingdom

### Publication situation

**Figure 5:**
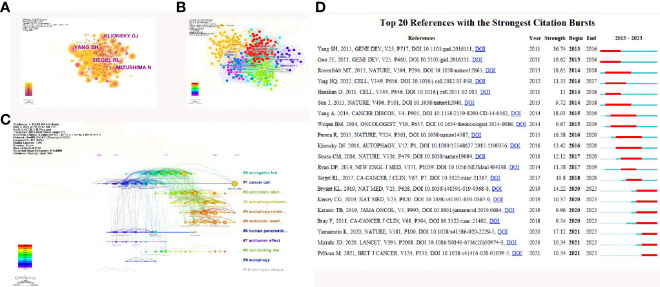
References related to the autophagy of pancreatic cancer. **(A)** Clustering analysis of the autophagy of the pancreatic cancer cocitation network drawn by CiteSpace. **(B)** Cluster view of co-citation references related to pancreatic cancer autophagy visualized by CiteSpace. **(C)** Timeline view of co-citation references related to pancreatic cancer autophagy visualized by CiteSpace. **(D)** The 20 references with the strongest citation bursts based on analysis in CiteSpace.

For the study of the autophagy of PC, among the top 10 most cited publications, five papers were in Autophagy (**Table 4**). Co-citations were first proposed as a measure of the relationship between publications by the American information scientist Small in 1973, which shows papers with a major impact on a particular field. The included papers cited a total of 64238 publications. The top N was set as 50 and the g-index factor κ was set as 15 in CiteSpace software, and a co-citation analysis of the publications was conducted and showed with abstract terms as labels (**Figure 5A**). Then the all in one clustering of co-citation of publications network was in progress and layed out in optimizing style (**Figure 5B**). The co-citation of publications network in time lines with the greatest impact were further analyzed (**Figure 5C**). Subsequently, the 20 references with the strongest citation bursts (**Figure 5D**) were obtained, which showed that the number of citations per period of a given paper increased rapidly, indicating that the contribution of each paper was relatively significant. In addition, the hot spot of quoting literature has gradually shifted from the study of autophagy-related pathways to ferroptosis, stellate cells, tumor microenvironment and the clinical transformation of the anti-tumor effect of inhibiting autophagy, which has received more attention in recent years.

**Table 4:** The 10 most cited articles regarding the autophagy of pancreatic cancer.

Title	Corresponding authors	Journal	Publication Year	Total Citations
Autophagy promotes ferroptosis by degradation of ferritin	Tang, Daolin	AUTOPHAGY	2016	848
Modulation of inflammation by autophagy: Consequences for human disease	Netea, Mihai G.	AUTOPHAGY	2016	237
MicroRNAs in apoptosis, autophagy and necroptosis	Yu, Qiang	ONCOTARGET	2015	268
Circular RNA IARS (circ-IARS) secreted by pancreatic cancer cells and located within exosomes regulates endothelial monolayer permeability to promote tumor metastasis	Li, Xiaowu	JOURNAL OF EXPERIMENTAL & CLINICAL CANCER RESEARCH	2018	219
Posttranslational modification of autophagy-related proteins in macroautophagy	Tang, Daolin	AUTOPHAGY	2015	211
Autophagy-dependent ferroptosis drives tumor-associated macrophage polarization *via* release and uptake of oncogenic KRAS protein	Tang, Daolin	AUTOPHAGY	2020	199
Inflammation, Autophagy, and Obesity: Common Features in the Pathogenesis of Pancreatitis and Pancreatic Cancer	Karin, Michael	GASTROENTEROLOGY	2013	198
MicroRNA 23b Regulates Autophagy Associated With Radioresistance of Pancreatic Cancer Cells	Meng, Zhiqiang	GASTROENTEROLOGY	2013	194
Cancer Development, Progression, and Therapy: An Epigenetic Overview	Tang, Daolin	INTERNATIONAL JOURNAL OF MOLECULAR SCIENCES	2013	176
Mitochondrial DNA stress triggers autophagy-dependent ferroptotic death	Jiang, Xuejun	AUTOPHAGY	2021	172

### Keyword analysis

**Figure 6:**
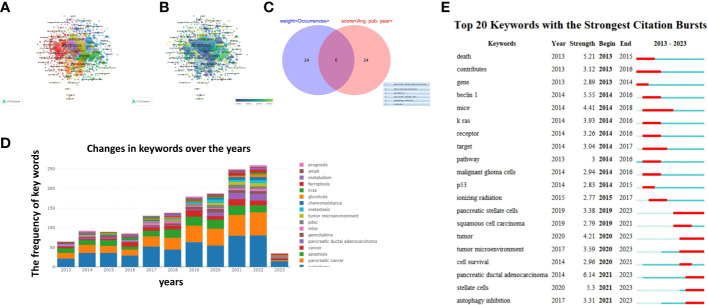
Keywords related to the autophagy of pancreatic cancer. **(A)** Network visualization of keywords drawn by VOSviewer. **(B)** Overlay visualization of keywords drawn by VOSviewer. **(C)** The autophagy effect study clusters according to the top 30 of weight <Occurrences> and score <Avg. pub. year>. **(D)** Changes in keywords over the years. **(E)** The 20 keywordss with the strongest citation bursts based on analysis in CiteSpace.

The keyword co-occurrence analysis by VOSviewer visually displayed keywords, which were clustered into three main categories. We summarize these three clusters as mechanisms of basic research (cluster 1, red), metabolic pathways (cluster 2, green), clinical application (cluster 3, blue) (**Figures 6A, B**). In cluster 1, the most recent three hot topics were oxidative stress (avg. pub. per year as of 2018.378, 85 occurrences), endoplasmic-reticulum stress (avg. pub. per year as of 2021.75,59 occurrences), epithelial-mesenchymal transition (avg. pub. per year as of 2018.386, 56 occurrences). The most recent three hot topics in cluster 2 were activation (avg. pub. per year as of 2018.2671, 147occurrences),metabolism(avg. pub. per year as of 2019.3229, 103 occurrences) and progression(avg. pub. per year as of 2019.2688, 91 occurrences).The most recent three hot topics in cluster 3 were apoptosis (avg. pub. per year as of 2018.3602, 352occurrences),gemcitabine(avg. pub. per year as of 2018.3289, 152 occurrences) and resistance(avg. pub. per year as of 2018.9158, 95 occurrences) (**Supplemental File 2**: **Table S1**)

### Dual-mapping overlay of the autophagy of PC

**Figure 7:**
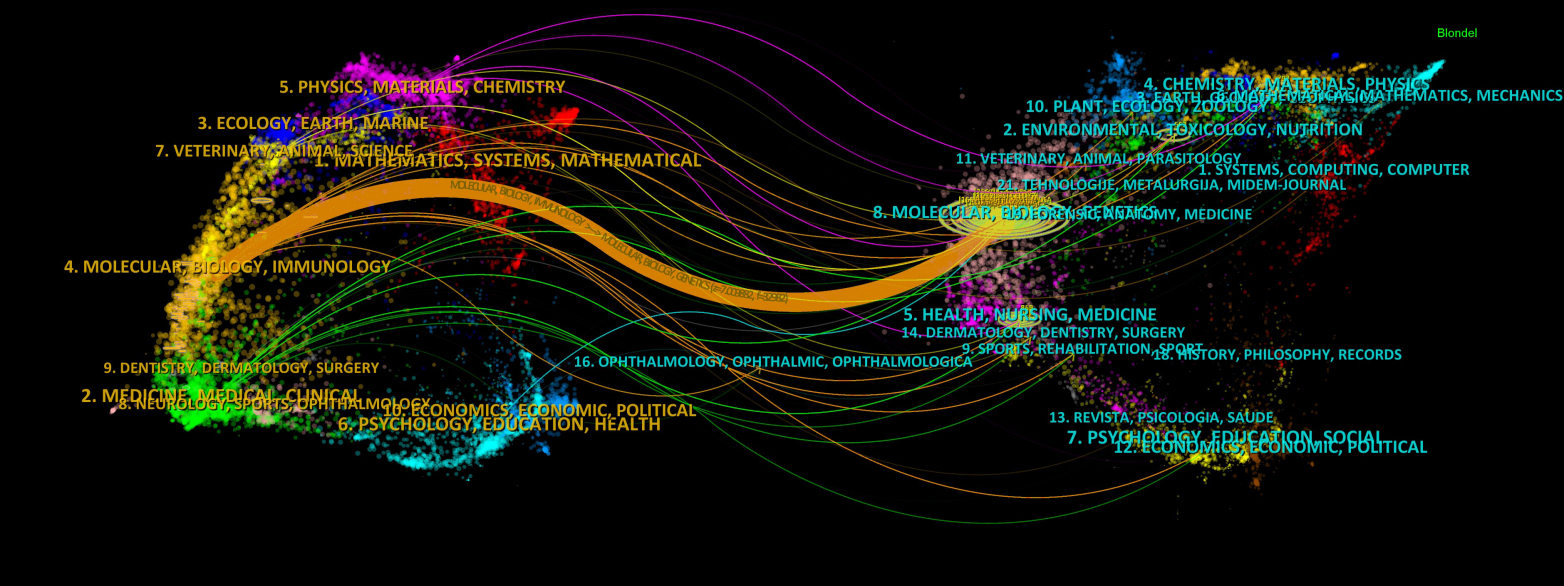
Dual-map overlay with publications on the autophagy of pancreatic cancer.

The dual-mapping overlay reveals the overall scientific contribution. The left side shows the citing journals; the right side represents the cited journals. The labels indicate the fields covered by the journal, the colored lines depict different citation paths, and the path width is proportional to the z-score scale. This connection illustrates the flow of knowledge and the relationship between different areas of research. We noted one main citation paths, the orange path, which indicated that articles from molecular, biology, and genetics journals were frequently cited by articles from molecular, biology, immunology (**Figure 7**).

### Clinical trials

Five clinical trials investigated the autophagy of PC between 2013 and 2023 (**Table 5**), with all from the USA. The three clinical trials mainly evaluated the efficacy and safety of drug hydroxychloroquine, which prevent autophagy, combinations with gemcitabine or binimetinib or Paricalcitol. Four clinical trials included more than 30 samples, which were in phase 2, the others were in phase 1.

**Table 5:** Clinical studies related to the autophagy of pancreatic cancer between 2013 and 2023.

Study	ClinicalTrials.gov Identifier	Official title	Time	Country	Design	No. of patients	Conditions	Intervention		Phase	Primary purpose
								Treatment group	Comparison group		
1	NCT04386057	LY3214996 +/- HCQ in Pancreatic Cancer	2020.12.13- 2023.12. 19	United States	Interventional	52	Pancreatic Cancer Advanced Cancer	Drug: Hydroxychloroquine Sulfate Drug: LY3214996	LY3214996	Phase 2	Treatment
2	NCT04132505	Binimetinib and Hydroxychloroquine in Treating Patients With KRAS Mutant metastatic Pancreatic Cancer	2019.10. 22 -2023.12.31	United States	Interventional	39	Metastatic Pancreatic AdenocarcinomaStage IV Pancreatic Cancer AJCC v8	Drug: Binimetinib Drug: Hydroxychloroquine	binimetinib	Phase 1	Treatment
3	NCT04524702	Paricalcitol and Hydroxychloroquine in Combination With Gemcitabine and Nab-Paclitaxel for Advanced Pancreatic Cancer	2020.9.14 -2024.8.14	United States	Interventional	21	Advanced Pancreatic Adenocarcinoma Metastatic Pancreatic Adenocarcinoma Stage IV Pancreatic Cancer AJCC v8	Drug: Gemcitabine Drug: Hydroxychloroquine Drug: Nab-paclitaxel	Drug: Nab-paclitaxel Drug: Paricalcitol	Phase 2	Treatment
4	NCT01978184	Randomized Phase II Trial of Pre-Operative Gemcitabine and Nab Paclitacel With or With Out Hydroxychloroquine	2013.11-2018.02.28	United States	Interventional	104	Pancreatic Cancer	Drug: gemcitabine Drug: abraxane Drug: hydroxychloroquine	Gemcitabine	Phase 2	Treatment
5	NCTNCT01506973	A Phase I/II/Pharmacodynamic Study of Hydroxychloroquine in Combination With Gemcitabine/Abraxane to inhibit Autophagy in Pancreatic Cancer	2011.12-2022.03	United States	Interventional	119	Advanced Adenocarcinoma Metastatic Adenocarcinoma	Drug: Hydroxychloroquine (HCQ) Drug: Gemcitabine Drug: Abraxane	Drug: Gemcitabine Drug: Abraxane	Phase1 Phase 2	Treatment

## Discussion

In this study, we used VOSviewer and CiteSpace software to conduct a visualization analysis of 1293 papers related to the autophagy of PC published between 2013 and March 28th 2023 to help researchers understand the current situation of global publications and predict research hotspots in future. From this study, the publication number and RRI for the autophagy of PC increased significantly, revealing that the popularity of research in this field has gradually increased (**Figure 3A**).

Over the past decade, there has been a yearly increase in publications related to autophagy research. Developed countries account for the majority of the top 20 countries/regions in terms of publications and collaborations with other countries/regions. The United States has the highest H-index (29), and a similar trend has been observed in autophagy-related research for other cancers (11, 27, 30, 31). There are 12 institutions in the United States that rank among the top 20 (**Table 1**), indicating a strong correlation between the country’s economic power and its scientific research (**Figure 3D**). As a developing country, China has experienced a rapid increase in the number of publications in this field, with only 13 in 2013. Since 2017, China’s annual publication count and total number of publications have surpassed those of the United States, making it the country with the most publications. In the field of cancer research, there has been a significant increase in the number of papers published by China in recent years (23, 24), which may be due to the fact that since 2010, cancer has become the leading cause of death in China, and the country has increased its investment in scientific research in public health and other fields in recent years (32, 33). Among the top 10 most cited papers, four are authored by American scholar Daolin Tang, who has achieved remarkable results in research on the regulation of autophagy and the effect of autophagy (**Table 4**), suggesting that this author wrote articles with greater academic impact. It is worth noting that although Chinese scholars have published the largest number of papers, their total citation count and average citation count are 17,298 and 28.77, respectively, with an H-index of 61. These citation numbers are relatively low, with only one of the top 10 most cited papers authored by a Chinese scholar, indicating that the quality of papers published by Chinese scholars needs further improvement.

The CiteSpace literature citation map and clusters demonstrate that the paper by YANG SH (34) has had a significant impact in the field (**Figures 5A–C**),when the prevailing view is environmental stress stimuli induced autophagy (35, 36). In this article, the authors proved that the development of pancreatic cancer exhibits a marked dependency on autophagy, with elevated levels of autophagy observed in primary pancreatic tumors and cell lines under basal conditions. Autophagy inhibition leads to metabolic defects in pancreatic cancer cells, ultimately resulting in significant growth suppression *in vitro*. More importantly, autophagy inhibition causes tumor regression and prolonged survival in pancreatic cancer xenografts and genetic mouse models. These findings suggest that autophagy is indeed essential for promoting tumorigenesis and growth of pancreatic cancer, and drugs that inactivate this process may have unique clinical utility in treating pancreatic cancer and other malignancies with similar autophagy dependency. Given that chloroquine and its derivatives are effective autophagy inhibitors and have been safely used in human patients for various purposes over decades, these results have been rapidly translated into relevant studies for pancreatic cancer treatment and have subsequently advanced to clinical trials (34).In the article by Mathias T. Rosenfeldt (37) and GUO JY (38)indicated Autophagy plays a crucial role in the metabolic reprogramming of tumors, including glucose uptake, synthetic metabolic pathways, the tricarboxylic acid (TCA) cycle, and energy expenditure during starvation. autophagy is required to sustain the functional mitochondrial pool necessary for tumor growth. These study makes targeting autophagy and mitochondrial metabolism valuable new approaches for treating aggressive pancreatic cancer (38).

These findings have provided considerable insights into the role of autophagy in cancer and established the significance of autophagy inhibition for cancer treatment. Currently, the lysosomal inhibitor hydroxychloroquine (HCQ) has been employed in numerous clinical trials, yielding mixed results (39). However, these findings have guided the development of more effective and specific autophagy inhibitors for use in clinical trials.

We also performed a timeline analysis of co-cited references to reveal the changes in research hotspots over time. The vertical axis represents the clustering label, with a total of 11 clusters, and the horizontal axis shows the timing of the occurrence of important reference nodes. The most recent research hotspots were “#5metabolic rewiring”and “#6 autophagy-related pathway” suggesting that concerns are not limited autophagy related genes of tumor, the breadth of research has been extended. (**Figure 5D**)

Keywords represent the main topic of publications. Through the co-citation analysis of VOSviewer, it is found that the whole study can be divided into three clusters: the regulation of autophagy cluster, the effect of autophagy cluster, and clinical study cluster (**Figures 6A, B**). In the regulation of autophagy cluster, the latest keywords focus on “LncRNA”, “circular RNA” and “miRNA” and other non-coding RNA.In recent years a number of studies have proved the role of non-coding RNA in the regulation of autophagy, the disorder between autophagy and non-coding RNA play a major role in the progress, treatment resistance of pancreatic cancer (PC), and the non-coding RNA as PC therapeutic target was explored (40–43).

The autophagy effect study clusters were intersection according to the top 30 of weight <Occurrences> and score <Avg. pub. year>,The six key words “autophagy inhibition”,”ferroptosis”,”pancreatic stellate cells”, “tumor microenvironment”, pancreatic ductal adenocarcinoma”, “lysosome “have received more attention in recent years and are the hot topics of this study (**Figure 6C**). Review and summary of relevant studies of the 6 keywords are shown that the autophagy not only help orchestrate the crosstalk of diverse vesicular trafficking pathways to remodel metabolism but also interface with multiple other cellular pathways, including (but not limited to)cell-death pathways, and innate immune signaling of tumor microenvironment (10). Ferroptosis is a novel form of cell death regulation, which Dr Stockwell BR found in a 2012 (44). It is characterized by metabolic abnormalities, leading to the accumulation of iron and related lipid signals, and lipid peroxidation produces reactive oxygen species, which induces cell death. The study of Daolin Tang et al. demonstrated that autophagy causes ferroptosis by degrading ferritin in cancer cells and fibroblasts in the microenvironment, clarifying the relationship between autophagy and ferroptosis at the gene level (45). Knockdown or knockout of Atg5 and Atg7 limited ferroptosis induced by erastin and reduced intracellular ferrous iron levels and lipid peroxidation. Consistently, inhibition of NCOA4 (nuclear receptor coactivator 4), a selective cargo receptor for selective autophagy turnover of ferritin (i. e., ferritin phagtosis) in ferroptosis. inhibits ferritin degradation and ferroptosis. In contrast, overexpression of NCOA4 increases ferritin degradation and promotes ferroptosis. These findings provide new insights into the interplay between autophagy and regulated cell death. Two other different studies show that MEKi in PDAC cells upregulates ferritin phagocytosis, which selectively degrades ferric protein from iron stores in the lysosomes to release iron in the cytosol. This increase in iron promotes mitochondrial iron-sulfur cluster in synthesis used to enhance mitochondrial oxidative phosphorylation in PDAC cells (46, 47). Targeted ferritin phagocytosis may be an interesting novel approach to inhibit autophagy, but more studies are needed.

The changes in malignant metabolism and malignant behavior of pancreatic cancer cells provide a suitable microenvironment for the survival of pancreatic cancer cells (25). pancreatic (PSCs) are an important component of the tumor microenvironment (48). PSCs are thought to play an important role in maintaining the balance between matrix formation and degradation and maintaining the normal tissue architecture, they can transfer into highly proliferative myofibroblasts in the presence of tissue injury (49). The confirmation of the presence of activated PSC in the stroma surrounding cancer cells has prompted investigation of possible interactions between these two cell types, and bidirectional interactions between PSC and cancer cells are now well reported [6]. PSCS can be activated in response to cancer cells (as demonstrated by increased proliferation, migration, and ECM synthesis); in turn, PSC induces cancer cell proliferation while reducing cancer cell apoptosis, thereby promoting cancer cell survival (50). PSC would also move cancer cells forward by affecting epithelial-mesenchymal transition (EMT) (51). Pancreatic ductal adenocarcinoma (PDAC) is characterized by a highly desmoplastic tumor microenvironment (TME) composed of a large number of activated PSCs, which play a key role in the refractory response of PDAC to immunotherapy and chemotherapy. In addition, several reports have shown that PSC induces resistance to radiotherapy and chemotherapy (52–54). PSCs promote cancer stem cell (CSC) to express stem-cell markers such as ABCG2, nestin, and Lin 28, which enabling CSCs to regenerate entire populations of cancer cells with varying degrees of differentiation that can serve as a source of recurrent, and commonly come into being tumors with therapy-resistant. Targeting tumor-stroma crosstalk in the tumor microenvironment has emerged as a promising therapeutic strategy against pancreatic cancer progression and metastasis. Vitamin D receptor signaling activation is a well-studied mechanism of PSC inactivation, and the vitamin D analogue calcipotriol (Cal) was found to activate VDR to induce aPSC quiescence. Synergistic autophagy blockade and VDR signaling activation enhance stellate cell reprogramming in PDAC in pancreatic ductal adenocarcinoma (55). Synergistic autophagy blockade and VDR signaling activation are promising therapeutic approaches to reprogram PSC and improve PDAC response to immunotherapy. Synergistic autophagy blockade and VDR signaling activation enhance stellate cell reprogramming in pancreatic ductal adenocarcinoma. And that idea has entered clinical trials. This phase 2 trial by Olatunji B. Alese et al. investigated the addition of paricalcitol and hydroxychloroquine to gemcitabine and nab-paclitaxel in patients with pancreatic cancer that had spread to other sites in the body (advanced or metastatic disease). Paricalcitol and hydroxychloroquine combined with standard chemotherapy (gemcitabine and albumin-bound paclitaxel) may be more effective than paricalcitol or hydroxychloroquine alone in patients with pancreatic cancer (NCT04524702); this trial will be completed in 2024 (56).

Another important cell type in the tumor microenvironment is cancer-associated fibroblasts (CAF), which are considered to be one of the most critical stromal cells that interact with pancreatic ductal adenocarcinoma (PDAC) and promote tumor growth, metastasis and treatment resistance. Previous studies have shown that autophagy contributes to CAF activation during tumor progression (53, 56).Defective autophagy in CAF has been found to impede CAF activation by inhibiting proline biosynthesis and collagen production. In addition, we show that autophagy promotes proline biosynthesis through mitophagy-mediated regulation of NADK 2 (mitochondrial NAD kinase 2), an enzyme responsible for mitochondrial NADP (H) production, Targeting PRKN (parkin RBR E3 ubiquitin protein ligase) in the matrix to inhibit cellular mitophagy reduced tumor weight. Therefore, inhibition of mitophagy in CAFs in the tumor microenvironment may be an attractive strategy to focus with stroma for anticancer intervention.

Autophagy inhibition in TME enhances anti-tumor immune response. Immunotherapy strategies aim to enhance anti-tumor immunity in cancer. However, clinical outcomes were less effective than expected. Recent findings suggest that autophagy inhibition not only in tumor cells but also in different cells present in the TME, such as T cells and tumor-associated macrophages (TAMs), can enhance anti-tumor immunity and enhance immune checkpoint inhibitor therapies, such as anti-programmed cell death protein I (PD-1) antibody therapy (57, 58). These findings highlight that autophagy in tumor cells and host cells supports tumor growth. TAMs is also an important component of TME, and autophagy inhibition in TAM promotes M1 polarization, leading to increased secretion of proinflammatory cytokines (59). In a melanoma model, HCQ treatment repolarized macrophages from M2 to M1 phenotype and also upregulated stimulator of interferon genes (STING) (60). Upregulated STING leads to phosphorylation of TBKl and release of interferon beta. Tam-induced interferon secretion enhanced tumor-directed cytotoxic T cells, and HCQ treatment also enhanced anti-PD-I antibody antitumor activity (59, 61). Together, these findings suggest that targeting autophagy in TAMs may be a promising approach and may open up the possibility of different drug delivery modalities for autophagy drugs, such as lipid nanoparticles that bind to macrophages (15). Natural killer (NK) cells and autophagy in dendritic cells (DC) have not been studied in pancreatic cancer, but PPTl deficient DC are more effective in cross-presenting viral antigens to T cells. Show that autophagy inhibition can strengthen the function of DC in the TME (58), whether can be used as effective inhibit autophagy immunotherapy targets remains to be further study.

The three most recent keywords in the clinical research cluster are “drug repurposing”, “immunotherapy”, and “drug repositioning”. It is also a research hotspot in clinical application in recent years. Patients with advanced cancer receive chemotherapy, targeted therapy or immunotherapy, but in most cases, these treatments are incurable (17). Autophagy promotes the survival and resistance of cancer cells in the face of chemotherapy and targeted therapy, and recent studies have also found its role in impairing anti-tumor immunity. The classical drug, chloroquine analogues, has entered several clinical trials for its new use as an autophagy inhibitor. However, inhibition of autophagy by HCQ alone or in combination is not curative in existing preclinical models and clinical trials, suggesting that there are mechanisms of tumor resistance to this approach. And precise delivery to tumor cells or microenvironment cells, improve the efficiency of traditional drugs and immunotherapy drugs, and reduce side effects, has become a hot topic for researchers (58). The poor effects of various current chemoradiotherapy and immunotherapy are closely related to micro environmental changes such as extensive fibrosis of PC caused by astrocytes. Combination of targeting of autophagy and astrocytes in PC may be an effective alternative to immune checkpoint therapy (54, 60). However, the five current clinical trials on autophagy in PC are mostly single-center clinical trials with small patient numbers, low evidence levels, and mainly phase I-II trials. These shortcomings may be related to the heterogeneity of PC itself, the diversity of dialogue between stromal cells such as astrocytes and tumor cells, and the complexity of the tumor microenvironment (23, 25, 26). To date, no specific autophagy inhibitors or inducers have received regulatory approval in cancer or any other disease. These questions raise the need to find better drugs to target autophagy in cancer. Therefore, it is promising to consider therapies that target autophagy, PSCs and tumor microenvironment, and a combination of classical antineoplastic agents. We expect that multidisciplinary integration can achieve more comprehensive research, resource sharing, and complementary advantages, so as to deepen the level of research and improve the effectiveness of pancreatic cancer treatment.

## Limitations

There are several limitations that should be considered in this study. We only searched the WOSCC database to ensure the quality and integrity of the collected data. The lack of a complement from other literature databases, such as Scopus or Google Scholar, may have led to an incomplete analysis of the data. Secondly, this study only analyzed basic publication information and lacked in-depth exploration of the specific content, which is somewhat subjective. Finally, we only selected articles published in English from 2013 to 2023. Some important and landmark studies may have been excluded.

## Conclusion

Pancreatic cancer is a severe disease with high morbidity and mortality. Moreover, the mechanism of the autophagy has extensive application prospects. The trend of the overall publications shows annual increases, indicating interest in this research filed is increasing. China has the most publications in this field, and publications are increasing at the fastest rate. Publications originating from the USA have the highest influence, which enlighten that it is necessary for countries to strengthen academic cooperation to help research progress. Ferroptosis, pancreatic stellate cells, tumor microenvironment, immunotherapy, and drug repositioning have been research hotspots in this field in recent years, which indicated that the current research hotspots mainly focus on the crosstalk between autophagy and pancreatic stellate cells or other tumor microenvironment component.

## Data availability statement

The raw data supporting the conclusion of the article will be made available by the authors, without due reservation.

## Author contributions

Study Concept: LF, HY. Manuscript Draft: ZW, LL, XQ. Data Collection: LF, HY. Data Analysis: ZW, LL, XQ. All authors contributed to the article and approved the submitted version.

## References

[1] Sung H Ferlay J Siegel RL Laversanne M Soerjomataram I Jemal A. Global cancer statistics 2020: GLOBOCAN estimates of incidence and mortality worldwide for 36 cancers in 185 countries. CA Cancer J Clin (2021) 71(3):209–49. doi: 10.3322/caac.21660 33538338

[2] Bear AS Vonderheide RH O'Hara MH. Challenges and opportunities for pancreatic cancer immunotherapy. Cancer Cell (2020) 38(6):788–802. doi: 10.1016/j.ccell.2020.08.004 32946773 PMC7738380

[3] Xie C Xu X Wang X Wei S Shao L Chen J. Cyclooxygenase-2 induces angiogenesis in pancreatic cancer mediated by prostaglandin E(2). Oncol Lett (2018) 16(1):940–8. doi: 10.3892/ol.2018.8786 PMC6019925 29963167

[4] Klionsky DJ Petroni G Amaravadi RK Baehrecke EH Ballabio A Boya P. Autophagy in major human diseases. EMBO J (2021) 40(19):e108863. doi: 10.15252/embj.2021108863 34459017 PMC8488577

[5] Tang R Xu J Zhang B Liu J Liang C Hua J. Ferroptosis, necroptosis, and pyroptosis in anticancer immunity. J Hematol Oncol (2020) 13(1):110. doi: 10.1186/s13045-020-00946-7 32778143 PMC7418434

[6] Tang D Chen X Kang R Kroemer G. Ferroptosis: molecular mechanisms and health implications. Cell Res (2021) 31(2):107–25. doi: 10.1038/s41422-020-00441-1 PMC8026611 33268902

[7] Xiao J Liu Z Wang J Zhang S Zhang Y. Identification of cuprotosis-mediated subtypes, the development of a prognosis model, and influence immune microenvironment in hepatocellular carcinoma. Front Oncol (2022) 12:941211. doi: 10.3389/fonc.2022.941211 36110946 PMC9468823

[8] Liu X Nie L Zhang Y Yan Y Wang C Colic M. Actin cytoskeleton vulnerability to disulfide stress mediates disulfidptosis. Nat Cell Biol (2023) 25(3):404–14. doi: 10.1038/s41556-023-01091-2 PMC10027392 36747082

[9] Mizushima N Komatsu M. Autophagy: renovation of cells and tissues. Cell (2011) 147(4):728–41. doi: 10.1016/j.cell.2011.10.026 22078875

[10] Levine B Kroemer G. Biological functions of autophagy genes: a disease perspective. Cell (2019) 176(1-2):11–42. doi: 10.1016/j.cell.2018.09.048 30633901 PMC6347410

[11] Li X He S Ma B. Autophagy and autophagy-related proteins in cancer. Mol Cancer. (2020) 19(1):12. doi: 10.1186/s12943-020-1138-4 31969156 PMC6975070

[12] Zhao YG Codogno P Zhang H. Machinery, regulation and pathophysiological implications of autophagosome maturation. Nat Rev Mol Cell Biol (2021) 22(11):733–50. doi: 10.1038/s41580-021-00392-4 PMC8300085 34302147

[13] Nakatogawa H. Mechanisms governing autophagosome biogenesis. Nat Rev Mol Cell Biol (2020) 21(8):439–58. doi: 10.1038/s41580-020-0241-0 32372019

[14] Yamamoto K Venida A Yano J Biancur DE Kakiuchi M Gupta S. Autophagy promotes immune evasion of pancreatic cancer by degrading MHC-I. Nature (2020) 581(7806):100–5. doi: 10.1038/s41586-020-2229-5 PMC7296553 32376951

[15] Li YJ Lei YH Yao N Wang CR Hu N Ye WC. Autophagy and multidrug resistance in cancer. Chin J Cancer. (2017) 36(1):52. doi: 10.1186/s40880-017-0219-2 28646911 PMC5482965

[16] Chen C Lu L Yan S Yi H Yao H Wu D. Autophagy and doxorubicin resistance in cancer. Anticancer Drugs (2018) 29(1):1–9. doi: 10.1097/CAD.0000000000000572 29099416

[17] Smith AG Macleod KF. Autophagy, cancer stem cells and drug resistance. J Pathol (2019) 247(5):708–18. doi: 10.1002/path.5222 PMC6668344 30570140

[18] Zamame Ramirez JA Romagnoli GG Kaneno R. Inhibiting autophagy to prevent drug resistance and improve anti-tumor therapy. Life Sci (2021) 265:118745. doi: 10.1016/j.lfs.2020.118745 33186569

[19] Ninkov A Frank JR Maggio LA. Bibliometrics: methods for studying academic publishing. Perspect Med Educ (2022) 11(3):173–6. 10.1007/s40037-021-00695-4 PMC9240160 34914027

[20] Wang H Shi J Shi S Bo R Zhang X Hu Y. Bibliometric analysis on the progress of chronic heart failure. Curr Probl Cardiol (2022) 47(9):101213. doi: 10.1016/j.cpcardiol.2022.101213 35525461

[21] Peng C Kuang L Zhao J Ross AE Wang Z Ciolino JB. Bibliometric and visualized analysis of ocular drug delivery from 2001 to 2020. J Control Release. (2022) 345:625–45. doi: 10.1016/j.jconrel.2022.03.031 35321827

[22] Song L Zhang J Ma D Fan Y Lai R Tian W. A bibliometric and knowledge-map analysis of macrophage polarization in atherosclerosis from 2001 to 2021. Front Immunol (2022) 13:910444. doi: 10.3389/fimmu.2022.910444 35795675 PMC9250973

[23] Wu K Liu Y Liu L Peng Y Pang H Sun X. Emerging trends and research foci in tumor microenvironment of pancreatic cancer: a bibliometric and visualized study. Front Oncol (2022) 12:810774. doi: 10.3389/fonc.2022.810774 35515122 PMC9063039

[24] Zhang Z Zhu Y Wang Q Chang T Liu C Zhu Y. Global trends and research hotspots of exercise for intervening diabetes: a bibliometric analysis. Front Public Health (2022) 10:902825. doi: 10.3389/fpubh.2022.902825 35875005 PMC9300903

[25] Xu Q Zhou Y Zhang H Li H Qin H Wang H. Bibliometric analysis of hotspots and frontiers of immunotherapy in pancreatic cancer. Healthcare (Basel). (2023) 11(3):304. doi: 10.3390/healthcare11030304 36766879 PMC9914338

[26] Sheahan AV Biankin AV Parish CR Khachigian LM. Targeted therapies in the management of locally advanced and metastatic pancreatic cancer: a systematic review. Oncotarget (2018) 9(30):21613–27. doi: 10.18632/oncotarget.25085 PMC5940404 29765563

[27] Shen J Shen H Ke L Chen J Dang X Liu B. Knowledge mapping of immunotherapy for hepatocellular carcinoma: a bibliometric study. Front Immunol (2022) 13:815575. doi: 10.3389/fimmu.2022.815575 35173728 PMC8841606

[28] Chen C Song M. Visualizing a field of research: a methodology of systematic scientometric reviews. PloS One (2019) 14(10):e0223994. doi: 10.1371/journal.pone.0223994 31671124 PMC6822756

[29] Ali MJ. Forewarned is forearmed: the h-index as a scientometric. Semin Ophthalmol (2021) 36(1-2):1. doi: 10.1080/08820538.2021.1894889 33734008

[30] Shi Y Wei W Li L Wei Q Jiang F Xia G. The global status of research in breast cancer liver metastasis: a bibliometric and visualized analysis. Bioengineered (2021) 12(2):12246–62. doi: 10.1080/21655979.2021.2006552 PMC8810156 34783637

[31] Ma L Ma J Teng M Li Y. Visual analysis of colorectal cancer immunotherapy: a bibliometric analysis from 2012 to 2021. Front Immunol (2022) 13:843106. doi: 10.3389/fimmu.2022.843106 35432385 PMC9009266

[32] Guan X Zhang Y Wushouer H Shi L Ross-Degnan D Wagner AK. Differences in reimbursement listing of anticancer therapies in China: an observational study. BMJ Open (2020) 10(1):e031203. doi: 10.1136/bmjopen-2019-031203 PMC6955534 31911513

[33] Wang L Wang Z Ma Q Fang G Yang J. The development and reform of public health in China from 1949 to 2019. Global Health (2019) 15(1):45. doi: 10.1186/s12992-019-0486-6 31266514 PMC6604346

[34] Yang S Wang X Contino G Liesa M Sahin E Ying H. Pancreatic cancers require autophagy for tumor growth. Genes Dev (2011) 25(7):717–29. doi: 10.1101/gad.2016111 PMC3070934 21406549

[35] Mizushima N Levine B Cuervo AM Klionsky DJ. Autophagy fights disease through cellular self-digestion. Nature (2008) 451(7182):1069–75. doi: 10.1038/nature06639 PMC2670399 18305538

[36] Sachdeva UM Thompson CB. Diurnal rhythms of autophagy: implications for cell biology and human disease. Autophagy (2008) 4(5):581–9. doi: 10.4161/auto.6141 18437053

[37] Rosenfeldt MT O'Prey J Morton JP Nixon C MacKay G Mrowinska A. p53 status determines the role of autophagy in pancreatic tumour development. Nature (2013) 504(7479):296–300. doi: 10.1038/nature12865 24305049

[38] Guo JY Chen HY Mathew R Fan J Strohecker AM Karsli-Uzunbas G. Activated ras requires autophagy to maintain oxidative metabolism and tumorigenesis. Genes Dev (2011) 25(5):460–70. doi: 10.1101/gad.2016311 PMC3049287 21317241

[39] Jain V Singh MP Amaravadi RK. Recent advances in targeting autophagy in cancer. Trends Pharmacol Sci (2023) 44(5):290–302. doi: 10.1016/j.tips.2023.02.003 36931971 PMC10106406

[40] Wu G Yu W Zhang M Yin R Wu Y Liu Q. MicroRNA-145-3p suppresses proliferation and promotes apotosis and autophagy of osteosarcoma cell by targeting HDAC4. Artif Cells Nanomed Biotechnol (2018) 46(sup2):579–86. doi: 10.1080/21691401.2018.1464459 29893594

[41] Hu Z Cai M Zhang Y Tao L Guo R. miR-29c-3p inhibits autophagy and cisplatin resistance in ovarian cancer by regulating FOXP1/ATG14 pathway. Cell Cycle (2020) 19(2):193–206. doi: 10.1080/15384101.2019.1704537 31885310 PMC6961660

[42] Zhou C Yi C Yi Y Qin W Yan Y Dong X. LncRNA PVT1 promotes gemcitabine resistance of pancreatic cancer *via* activating wnt/β-catenin and autophagy pathway through modulating the miR-619-5p/Pygo2 and miR-619-5p/ATG14 axes. Mol Cancer. (2020) 19(1):118. doi: 10.1186/s12943-020-01237-y 32727463 PMC7389684

[43] He Z Cai K Zeng Z Lei S Cao W Li X. Autophagy-associated circRNA circATG7 facilitates autophagy and promotes pancreatic cancer progression. Cell Death Dis (2022) 13(3):233. doi: 10.1038/s41419-022-04677-0 35288538 PMC8921308

[44] Dixon SJ Lemberg KM Lamprecht MR Skouta R Zaitsev EM Gleason CE. Ferroptosis: an iron-dependent form of nonapoptotic cell death. Cell (2012) 149(5):1060–72. doi: 10.1016/j.cell.2012.03.042 PMC3367386 22632970

[45] Hou W Xie Y Song X Sun X Lotze MT Zeh HJ 3rd. Autophagy promotes ferroptosis by degradation of ferritin. Autophagy (2016) 12(8):1425–8. doi: 10.1080/15548627.2016.1187366 PMC4968231 27245739

[46] Datta J Dai X Bianchi A De Castro Silva I Mehra S Garrido VT. Combined MEK and STAT3 inhibition uncovers stromal plasticity by enriching for cancer-associated fibroblasts with mesenchymal stem cell-like features to overcome immunotherapy resistance in pancreatic cancer. Gastroenterology (2022) 163(6):1593–612. doi: 10.1053/j.gastro.2022.07.076 PMC10257389 35948109

[47] Nagathihalli NS Castellanos JA Lamichhane P Messaggio F Shi C Dai X. Inverse correlation of STAT3 and MEK signaling mediates resistance to RAS pathway inhibition in pancreatic cancer. Cancer Res (2018) 78(21):6235–46. doi: 10.1158/0008-5472.CAN-18-0634 PMC6878978 30154150

[48] Apte MV Pirola RC Wilson JS. Pancreatic stellate cells: a starring role in normal and diseased pancreas. Front Physiol (2012) 3:344. doi: 10.3389/fphys.2012.00344 22973234 PMC3428781

[49] Hamada S Masamune A Takikawa T Suzuki N Kikuta K Hirota M. Pancreatic stellate cells enhance stem cell-like phenotypes in pancreatic cancer cells. Biochem Biophys Res Commun (2012) 421(2):349–54. doi: 10.1016/j.bbrc.2012.04.014 22510406

[50] Sousa CM Biancur DE Wang X Halbrook CJ Sherman MH Zhang L. Pancreatic stellate cells support tumour metabolism through autophagic alanine secretion. Nature (2016) 536(7617):479–83. doi: 10.1038/nature19084 PMC5228623 27509858

[51] Kikuta K Masamune A Watanabe T Ariga H Itoh H Hamada S. Pancreatic stellate cells promote epithelial-mesenchymal transition in pancreatic cancer cells. Biochem Biophys Res Commun (2010) 403(3-4):380–4. doi: 10.1016/j.bbrc.2010.11.040 21081113

[52] Gore J Korc M. Pancreatic cancer stroma: friend or foe? Cancer Cell (2014) 25(6):711–2. doi: 10.1016/j.ccr.2014.05.026 PMC4821630 24937454

[53] Hwang RF Moore T Arumugam T Ramachandran V Amos KD Rivera A. Cancer-associated stromal fibroblasts promote pancreatic tumor progression. Cancer Res (2008) 68(3):918–26. doi: 10.1158/0008-5472.CAN-07-5714 PMC2519173 18245495

[54] Hwang HJ Oh MS Lee DW Kuh HJ. Multiplex quantitative analysis of stroma-mediated cancer cell invasion, matrix remodeling, and drug response in a 3D co-culture model of pancreatic tumor spheroids and stellate cells. J Exp Clin Cancer Res (2019) 38(1):258. doi: 10.1186/s13046-019-1225-9 31200779 PMC6567511

[55] Sherman MH Yu RT Engle DD Ding N Atkins AR Tiriac H. Vitamin d receptor-mediated stromal reprogramming suppresses pancreatitis and enhances pancreatic cancer therapy. Cell (2014) 159(1):80–93. doi: 10.1016/j.cell.2014.08.007 25259922 PMC4177038

[56] Bai J Liu T Tu B Yuan M Shu Z Fan M. Autophagy loss impedes cancer-associated fibroblast activation *via* downregulating proline biosynthesis. Autophagy (2023) 19(2):632–43. doi: 10.1080/15548627.2022.2093026 PMC9851237 35786294

[57] Ou P Wen L Liu X Huang J Huang X Su C. Thioesterase PPT1 balances viral resistance and efficient T cell crosspriming in dendritic cells. J Exp Med (2019) 216(9):2091–112. doi: 10.1084/jem.20190041 PMC6719428 31262842

[58] Sharma G Ojha R Noguera-Ortega E Rebecca VW Attanasio J Liu S. PPT1 inhibition enhances the antitumor activity of anti-PD-1 antibody in melanoma. JCI Insight (2020) 5(17):e133225. doi: 10.1172/jci.insight.133225 32780726 PMC7526447

[59] Chen D Xie J Fiskesund R Dong W Liang X Lv J. Chloroquine modulates antitumor immune response by resetting tumor-associated macrophages toward M1 phenotype. Nat Commun (2018) 9(1):873. 29491374 10.1038/s41467-018-03225-9 PMC5830447

[60] Luo X Qiu Y Dinesh P Gong W Jiang L Feng X. The functions of autophagy at the tumour-immune interface. J Cell Mol Med (2021) 25(5):2333–41. doi: 10.1111/jcmm.16331 PMC7933948 33605033

[61] Noman MZ Parpal S Van Moer K Xiao M Yu Y Viklund J. Inhibition of Vps34 reprograms cold into hot inflamed tumors and improves anti-PD-1/PD-L1 immunotherapy. Sci Adv (2020) 6(18):eaax7881. doi: 10.1126/sciadv.aax7881 32494661 PMC7190323

